# Influence of Green Tides in Coastal Nursery Grounds on the Habitat Selection and Individual Performance of Juvenile Fish

**DOI:** 10.1371/journal.pone.0170110

**Published:** 2017-01-26

**Authors:** Emilie Le Luherne, Olivier Le Pape, Laurence Murillo, Marine Randon, Clément Lebot, Elodie Réveillac

**Affiliations:** 1 ESE, Ecology and Ecosystems Health, Agrocampus Ouest, INRA, Rennes, France; 2 LIttoral ENvironnement et Sociétés (LIENSs), UMR 7266 CNRS-Université de La Rochelle, Institut du Littoral et de l'Environnement, 2, rue Olympe de Gouges, La Rochelle, France; Department of Agriculture and Water Resources, AUSTRALIA

## Abstract

Coastal ecosystems, which provide numerous essential ecological functions for fish, are threatened by the proliferation of green macroalgae that significantly modify habitat conditions in intertidal areas. Understanding the influence of green tides on the nursery function of these ecosystems is essential to determine their potential effects on fish recruitment success. In this study, the influence of green tides on juvenile fish was examined in an intertidal sandy beach area, the Bay of Saint-Brieuc (Northwestern France), during two annual cycles of green tides with varying levels of intensity. The responses of three nursery-dependent fish species, the pelagic *Sprattus sprattus* (L.), the demersal *Dicentrarchus labrax* (L.) and the benthic *Pleuronectes platessa* L., were analysed to determine the effects of green tides according to species-specific habitat niche and behaviour. The responses to this perturbation were investigated based on habitat selection and a comparison of individual performance between a control and an impacted site. Several indices on different integrative scales were examined to evaluate these responses (antioxidant defence capacity, muscle total lipid, morphometric condition and growth). Based on these analyses, green tides affect juvenile fish differently according to macroalgal density and species-specific tolerance, which is linked to their capacity to move and to their distribution in the water column. A decreasing gradient of sensitivity was observed from benthic to demersal and pelagic fish species. At low densities of green macroalgae, the three species stayed at the impacted site and the growth of plaice was reduced. At medium macroalgal densities, plaice disappeared from the impacted site and the growth of sea bass and the muscle total lipid content of sprat were reduced. Finally, when high macroalgal densities were reached, none of the studied species were captured at the impacted site. Hence, sites affected by green tides are less favourable nursery grounds for all the studied species, with species-specific effects related to macroalgal density.

## Introduction

Coastal areas are productive systems that encompass essential habitats, such as nursery grounds, for various fish species [1,2], including many economically valuable species [3]. The recruitment success of these fish species is highly dependent on the quality of coastal nurseries, which modulate the growth, condition and survival of juvenile fish [4–6]. The suitability of these sensitive habitats is threatened by numerous anthropogenic pressures [7], including massive seasonal proliferations of free-floating green macroalgae, called green tides. This form of eutrophication has spread along many coastlines and has increased in occurrence, abundance and duration worldwide since the 1970s [8–10]. The proliferation of green macroalgae leads to major changes in habitat structure [11–13], water chemistry and biogeochemical cycles [14,15]. These changes disturb ecological communities [13,16] and affect both trophic food webs and ecosystem processes [17–19]. Thus, abiotic and biotic modifications linked to green tides could affect the habitat suitability for coastal nursery-dependent fish species [13,20–22]. The consequences of these changes for fish species are modulated by the composition, intensity and duration of the macroalgal bloom [23,22]. Patchy or weak macroalgal proliferation could be beneficial to juvenile marine fish by providing new food resources and new shelter on unvegetated substrates [24–26]. Conversely, high and long-term proliferation could be detrimental for fish [20,21], probably as a result of reduced foraging efficiency [27], and could even lead to the total disappearance of fish from impacted sites [22]. The modification of habitat conditions caused by green tides and the response of fish communities have been previously described [13,20,22]. However, the underlying ecological processes, especially the impacts of green tides on habitat selection and individual performance for various nursery-dependent fish species requires investigation [23].

In Northwestern France, many shallow sandy beaches experience seasonal proliferations of green macroalgae. Among them, the Bay of Saint-Brieuc is the most heavily impacted by green tides [28]. Each year, the intertidal area of this coastal bay is covered by green macroalgae that develops in free-floating expanded blade form (mostly *Ulva armoricana* and *U*. *rotundata*) from spring to the end of summer [22,28–30]. In temperate latitudes, this corresponds with the settlement and growth period of juvenile marine fish in shallow coastal areas [4,22,31]. To analyse the impact of green tides on the nursery-dependent fish species that gather in the Bay of Saint-Brieuc, three teleost fish species with different vertical distributions, and thus potentially different responses to green tides [22], were examined: a pelagic species, the European sprat *Sprattus sprattus* (Linnaeus, 1758); a demersal species, the European sea bass *Dicentrarchus labrax* (Linnaeus, 1758); and a benthic species, the European plaice *Pleuronectes platessa* Linnaeus, 1758. The effects of green tides on these three species were evaluated by comparing, for each species, behaviour and individual performance at an impacted and a control site during two annual cycles. Initially, the influence of green tides on habitat selection was examined based on the combination of fish density and habitat-specific stable isotope signature in fish muscle [32,33]. Then, physiological adjustments were analysed for individuals who lived in coastal areas during green macroalgae proliferations. Instantaneous to mid-term physiological responses of fish to green tides were assessed using fish antioxidant defence capacity [34], muscle total lipid [35–37], morphometric condition [38] and daily growth rate [39,40]. These responses were analysed with respect to the period and intensity of green tides and the life history of the three studied fish species. We examined several factors to improve the understanding of the influence of green tide on fish at the individual scale, focusing on: (i) different integrative scales of perturbation; (ii) macroalgae density and (iii) species specific ecological niche and behaviour.

## Materials and Methods

### Ethics statement

Permission to collect fish with a trawl net in the study areas was granted by the French Departmental Authority for Maritime Affairs “Direction Départementale des Territoires et de la Mer (DDTM) des Côtes d’Armor” (ddtm-dml@cotes-darmor.gouv.fr) after examination of the sampling protocol. Surveys in the impacted area were performed in a marine protected area, the National Nature Reserve of Saint-Brieuc. Sampling was conducted in full agreement and in collaboration with the reserve managers. In accordance with European Commission recommendation 2007/526/EC, on revised guidelines for the accommodation and care of animals used for experimental and other scientific purposes, fish sampling in the wild without experimental handling did not require an ethics agreement. Fish caught were immediately immersed in ice to be sacrificed by hypothermia in the field. The present field study did not involve endangered or protected species.

### Selection of the study sites

**Fig 1:**
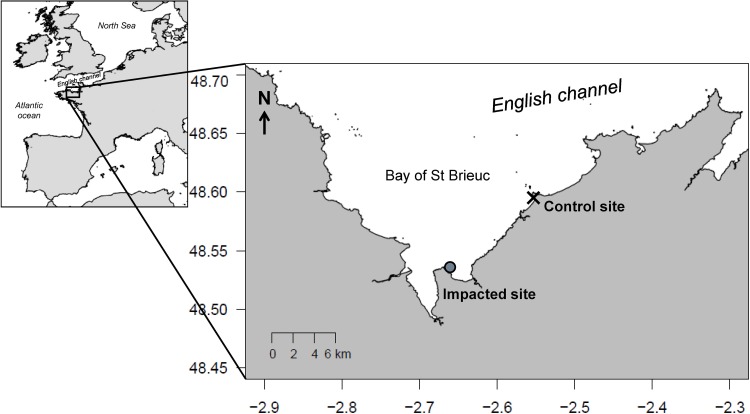
Location of the control (C) and impacted (I) sites in the study area, Northwestern France.

To examine the effects of green tides on fish habitat selection and individual performance, a control (48°35.7’N, 2°33.3’W) and an impacted (48°31.9’N, 2°39.7’W) site were selected in the Bay of Saint-Brieuc (Fig 1). Sites were chosen for their similarity in sediment structure and their proximity (10 km), to be comparable in terms of larval supply and habitat suitability [22]. Moreover, the 10 km distance was considered sufficient to prevent significant movement of juveniles of the studied fish species between sites [41–43], avoiding potential mixing.

### Field sampling

The sampling design was based on a standardised field survey performed in 2013 and 2014 from April until October to investigate the response of the fish community to green tides [22]. At both the control and impacted sites, sampling was conducted twice a month at the beginning of ebb tide in the shallow upper intertidal zone. At each sampling date and site, 6 successive trawl hauls of 25 m were conducted with a trawl net (mesh of 8 mm) towed by two people at a depth between 0.4 and 0.7 m. An additional sampling session was conducted over a 24 h period in late spring in 2014 [22]. The biomass of expanded blade *Ulva* spp. towed in each trawl haul was weighed as the wet weight after one minute of draining. Simultaneously, seawater physico-chemical parameters (i.e., temperature (°C), salinity, pH and dissolved oxygen concentration (mg.L^-1^)) were measured in the middle of the water column using a multiparameter probe (Hanna HI 9828/4-02).

For the three selected species, fish were collected and stored in coolers in the field (for a one to three hour period). Fish condition (i.e., unresponsive or dead individuals) was verified for each individual prior freezing in coolers containing ice and transportation to the laboratory. At the laboratory, they were measured (total length to the nearest mm) and weighed (total mass to the nearest mg), before being individually frozen at -20°C for further analysis. For each fish species, only sampling dates that collected at least 3 individuals at both sites after the onset of the green tide were retained for analyses on the individual scale. In addition, to test the activation of antioxidant defence capacity in response to green tides, a specific protocol was set. Fish of each species were collected on one date during macroalgae proliferation (S1 Table) and were deep-frozen individually in the field at -80°C in liquid nitrogen.

### Selection of fish for analysis

Green tides induced a drastic decrease in fish density at different levels of macroalgal density specific to each species, until the total disappearance of fish during the period of maximum macroalgae density [22]. For each species and each year, the sampling allowed the examination of fish response during green tides for a restricted number of sampling dates that differed between species (Table 1 and S1 Table).

**Table 1:** Number (N) of fish analysed and their total length range (TL in mm) at the control and impacted sites with the corresponding number of sampling dates (N of dates) analysed for the sprat (*S*. *sprattus*), sea bass (*D*. *labrax*) and plaice (*P*. *platessa*) in 2013 and 2014. For each index, “integration” refers to the range of its time of integration (or turnover rate).

Type of analysis	Integration	Species	Year	Site	N	TL (mm)	N of
							dates
δ^13^C signature	Month	Sprat	2013	Control	25	[35–47]	5
				Impacted	19	[33–47]	5
			2014	Control	25	[30–41]	5
				Impacted	23	[27–46]	5
		Sea bass	2014	Control	40	[28–135]	6
				Impacted	35	[26–134]	6
		Plaice	2013	Control	5	[36–52]	1
				Impacted	5	[33–45]	1
			2014	Control	11	[33–46]	1
				Impacted	6	[24–46]	1
Antioxidant	Hour	Sprat	2013	Control	5	[36–47]	1
defence capacity				Impacted	5	[33–47]	1
			2014	Control	10	[34–57]	1
				Impacted	11	[30–63]	1
		Sea bass	2014	Control	8	[109–135]	1
				Impacted	6	[86–133]	1
		Plaice	2014	Control	6	[65–78]	1
				Impacted	7	[55–71]	1
C:N ratio	≤ Week	Sprat	2013	Control	15	[36–47]	3
(muscle total lipid)				Impacted	12	[33–47]	3
			2014	Control	25	[30–41]	5
				Impacted	23	[27–46]	5
		Sea bass	2014	Control	26	[62–135]	3
				Impacted	19	[53–134]	3
		Plaice	2013	Control	5	[36–52]	1
				Impacted	5	[33–45]	1
			2014	Control	11	[33–46]	1
				Impacted	6	[24–46]	1
R morphometric	Weeks	Sprat	2013	Control	99	[35–46]	2
condition				Impacted	67	[32–49]	2
			2014	Control	501	[32–71]	5
				Impacted	312	[31–77]	5
		Sea bass	2014	Control	204	[25–141]	6
				Impacted	215	[23–134]	6
		Plaice	2013	Control	9	[25–73]	1
				Impacted	6	[33–45]	1
			2014	Control	32	[36–75]	1
				Impacted	7	[31–76]	1
Daily growth	≤ Week	Sprat	2013	Control	17	[35–41]	4
rate				Impacted	22	[32–44]	5
			2014	Control	23	[30–41]	5
				Impacted	22	[27–46]	5
		Sea bass	2014	Control	17	[28–75]	4
				Impacted	17	[26–56]	5
		Plaice	2013	Control	5	[36–52]	1
				Impacted	5	[33–45]	1
			2014	Control	8	[36–75]	1
				Impacted	7	[31–76]	1

All the selected fish were used for the R relative morphometric condition analysis (Table 1). For the δ^13^C signature, C:N ratio and daily growth rate (DGR) analyses, sub-samples of three to five individuals representative of the most frequent size class at both sites were selected for each sampling date studied (Table 1). For Trolox equivalent antioxidant capacity (TEAC) analyses, all deep-frozen fish within the same length class were analysed (Table 1).

### Antioxidant defence capacity

Antioxidant defence implemented to counteract oxidative stress [44] involves several mechanisms with species-specific patterns of activation [45]. Fish total antioxidant defence capacity was assessed by measuring the TEAC (mM Trolox equivalent). TEAC measurements were performed using the Antioxidant Assay Kit (AA Kit) (Sigma-Aldrich®—St Louis, USA) and following the kit procedure. Analyses were performed on a 50 mg piece of dorsal white muscle diluted in 1 mL of 1x Assay buffer. In parallel, the protein concentration (mg of soluble protein.mL^−1^) was measured in accordance with the manufacturer's instructions using the Bicinchoninic Acid Protein Assay Kit with Bovine Serum Albumin as standard (Sigma-Aldrich®—St Louis, USA). Prior to measuring the protein concentration, the sample solutions used for the TEAC measurements were diluted 1 in 5 with the 1x Assay buffer of the AA Kit to be in the range of protein concentrations covered by this method of analysis. The compatibility between reagents of the two kits was ensured by Sigma-Aldrich® Company. Measurements of both TEAC and soluble proteins were performed with a spectrofluorimeter (SAFAS Flx-Xenius, Monaco). The measured amounts of TEAC were proportional to the muscle soluble proteins released by fish muscle grinding and the TEAC results were thus expressed in mM Trolox equivalent/mg of soluble protein. Triplicate measurements were performed for the two biochemical analyses, and antioxidant defence capacity was analysed based on the individual mean of the values. Antioxidant defence capacity results were considered in relation to habitat conditions simultaneously recorded as their turnover rates are of one to a few hours for juvenile fish [45,46].

### Stable isotope analysis

Dorsal white muscle samples were freeze-dried, ground and encapsulated in tin cups to be analysed with a continuous-flow isotope-ratio mass spectrometer (Delta V Plus, Thermo Scientific) coupled to an elemental analyser (Flash 2000, Thermo Scientific). Two indices were derived from these analyses.

Firstly, the fish δ^13^C signature was used to examine fish habitat fidelity [32]. The δ^13^C signatures were expressed as isotope ratios relative to the international standard (i.e., VPDB: Vienna Pee Dee Belemnite; [47]) using the following formula:

$$\delta {}^{13}C\;(\text{in}\;‰)=\left[\frac{\left({}^{13}C:{}^{12}C_{sample}\right)}{\left({}^{13}C:{}^{12}C_{VPDB}\right)}-1\right]\times 10^{3}$$

Three characteristics of this marker make it useful for discriminating between the impacted and control sites: (i) the δ^13^C signature of an organism is related to the signature of its prey [48]; (ii) terrestrial primary producers have lower δ^13^C than marine producers [32,49], so the δ^13^C of fish captured at the impacted site (in the same marine water mass but closer to river inputs) should be lower than those of fish at the control site [50]; and (iii) the presence of large amounts of green macroalgae influence the δ^13^C of particulate organic matter (POM) at the base of the food web and this modification of δ^13^C propagates along the food web [33]. The invertebrate prey of juvenile fish have δ^13^C signatures that are approximately 1‰ higher at sites impacted by green tides than at control sites [33].

Based on these three characteristics, the spatiotemporal patterns in fish δ^13^C were used to trace fish habitats. Under green tides conditions, the higher fish δ^13^C signature at the impacted than at the control site reveals the fidelity of fish to the restricted area impacted by green tides. The turnover rates of the δ^13^C signatures in the white muscle of juvenile fish, revealed by experimental and field studies, reach 3 weeks on average [32,51–53] and 2 months for some species [54]. Hence, as the δ^13^C signatures represent an integration of habitat use during a time period linked to the turnover rate, changes in the kinetics of fish δ^13^C signatures were analysed according to habitat conditions at the same date but also for 2 earlier sampling dates (i.e., approximately 30 days earlier).

In addition, the C:N ratio was assessed to estimate fish muscle lipid content [35,36,55]. The C:N ratios were calculated using the following formula:

$$C:N_{\;\text{ratio}}=\frac{\%\;C}{\%\;N}$$

where %C = ^13^C/(^13^C+^12^C) and %N = ^15^N/(^15^N+^14^N). C:N ratios are expressed as the mass ratio.

A preliminary delipidation was performed based on the protocol developed by Chouvelon et al. [56] to measure fish muscle basal signature (i.e., without lipid; [57]). This analysis allowed us to validate the independence between fish length and fish basal signature for each species at the studied length range (S2 Table). The fish muscle total lipid content was analysed using C:N ratios measured on non-delipidated muscle as a proxy. The turnover rate of muscle total lipid for juvenile fish has been estimated to be less than a week [58]. Fish muscle total lipid were thus analysed in relation to the habitat conditions simultaneously recorded.

### Morphometric condition–R relative morphometric condition

R is a length-independent relative body condition index that examines the deviation of observed mass from predicted mass (using the log length-mass relationship). It is computed as follows [38]:

R=logW−logWc

where *W* is the observed total body mass and *Wc* is the computed body mass derived from the log length-mass relationships. Log length-mass relationships were established by species for each studied year based on all the individuals caught (S3 Table).

The R morphometric condition index was preferred to the widely used K Fulton’s condition index as the linear relation between fish mass and the cube of its length assumed by K Fulton’s index was not systematically verified for juvenile fish (S3 Table and [59–61]). The time of response of the morphometric conditions to environmental and food conditions has been established in a range of one to two weeks by experimental starvation of juvenile fish [62]. Fish morphometric conditions were thus analysed in relation to the habitat conditions simultaneously recorded.

### Daily growth rates (DGRs)

Prior to the use of otolith DGRs as a proxy for fish somatic growth, their daily deposition and the relation between otolith and somatic growth rates were verified for sprat [40], sea bass [63,64] and plaice [65,66]. Left sagittae were extracted, cleaned and mounted on a microscope slide with Crystal Bond® thermoplastic glue, sulcus side up for sprat and sulcus side down for sea bass and plaice. They were polished on a sagittal plane using a polishing pad (grit 2400) with distilled water until the microincrements could be read. Photographs were taken under a microscope using multiple magnifications (40x and 100x) with a Zeiss Axiocam ERc 5s® digital camera and ZEN 2012 (blue edition)® software (Carl Zeiss Microscopy GmbH). Photographs were compiled and counts of daily increments were performed randomly three times each by at least two independent observers. Counts of daily increments were made from the first to the last increment deposited at the outer edge of the otolith. The coefficient of variation (CV) of the counts was calculated for each otolith to quantify inconsistencies between readers [67]. When this CV was greater than 7%, otoliths were excluded from the analysis [67]. Using this selection process, 10 sprat otoliths, 2 sea bass otoliths and 1 plaice otolith were excluded from the analysis. When the CV was less than 7%, we used the mean of the three readings as an estimation of fish age. The estimated age and the date of fish capture were then used to back-calculate the date of the first increment deposition for each individual.

For each species, individuals were grouped by cohort based on the date of deposition of the first increment [68]. This grouping was performed to identify fish that originated from the same larval pool [69–71] and that experienced comparable environmental conditions throughout their development. Each cohort pooled a minimum of 3 individuals at each site. Within the same cohort, individuals had their first increment dates within one month.

To study the juvenile phase, the age and the date of fish settlement in coastal nurseries were acquired by counts from otolith post-larval settlement marks. These marks are delineated by the initiation of the last accessory growth centre for sea bass and plaice at the end of metamorphosis [63,72] and by the peak of microincrement width for sprat [60].

For each otolith, DGRs (in μm.d^-1^) were measured on a 5 increment interval basis (i.e., mean DGRs recorded over 5 days), once from the first increment and once from the settlement mark. These measurements were performed along the nucleus-post-rostrum axis for sprat and plaice and along the nucleus-dorsal axis for sea bass using ImageJ® software. Each measured DGR was associated with a fish, belonging to one cohort at one site, and with the starting date of a 5 day period.

Measurements from the first increment were plotted to visualise the DGRs of each cohort between sites from birth to capture. Then, for each of the three species, patterns of juvenile growth from the earliest date of settlement to the latest date of capture of the fish composing each cohort were statistically compared among sites. To compare intra-cohort DGRs between sites during juvenile growth, the period was split into 10-day sections, starting from the first day of the delineated juvenile period (i.e., the cohort settlement). This time step of 10 days fits the range of otolith integration during habitat condition modification [61,62,73] and enables the analysis of pooled DGRs according to the intensities of green macroalgae recorded in surveys, with a 2 week time step.

These 10-day sections were further considered as the factor “date” for the DGRs analysis. With regard to green tides, three situations existed for each 10-day section, i.e., *Before*, *During* and *After* green tides. Each “date” was thus associated with one green-tide-related situation and considered as a categorical factor.

### Statistical analyses

All the statistical analyses were performed using R version 3 [74], and significance was determined at the α = 0.05 level. The normality of the data distribution and the homoscedasticity of the variance were tested using the Shapiro-Wilk and Bartlett tests, respectively. The results were non-significant, which indicated that parametric statistical tests could be performed.

Linear models were used to test the influence of fish length on the TEAC, δ^13^C signature, C:N ratio and R morphometric condition. For all the studied species, fish length did not influence the indices at the selected length range. Therefore, statistical analyses were performed without preliminary correction. For each species and each year, potential differences between the control and impacted sites at the selected sample dates were examined. We applied one-way ANOVA when indices were assessed on a single date and two-way ANOVA when several dates were used (Table 1).

For each species and each year, and for each cohort identified from fish otoliths, each green tide period experienced throughout the juvenile stage (i.e., before, during and after proliferation) was considered. Potential differences in fish DGRs between the control and impacted sites were examined by one-way ANOVA when the DGR was assessed on a single date and by two-way ANOVA when several dates were assessed.

## Results

### Physico-chemical conditions

During each studied year, the seasonal cycles of salinity, pH and temperature did not vary between the control and impacted sites. This similarity across sites was validated by the daily variations recorded during the 24 h survey. However, the temperature range varied between years, i.e., between 8°C and 22°C in 2013 and 11°C and 21°C in 2014.

Apart from mid-June to mid-July 2013, when oxygen supersaturation (≥10 mg.L^-1^) was measured at the impacted site under high densities of *Ulva* spp., the daytime mean dissolved oxygen concentrations did not differ between sites (seasonal range from 7 to 10 mg.L^-1^). When measured throughout a 24 h cycle at a medium density of green macroalgae, the daily range had a larger amplitude at the impacted site, from 7 to 16 mg.L^-1^ (reaching oxygen supersaturation), than simultaneously recorded at the control site, 7 to 9 mg.L^-1^.

### Macroalgal cycles

**Fig 2:**
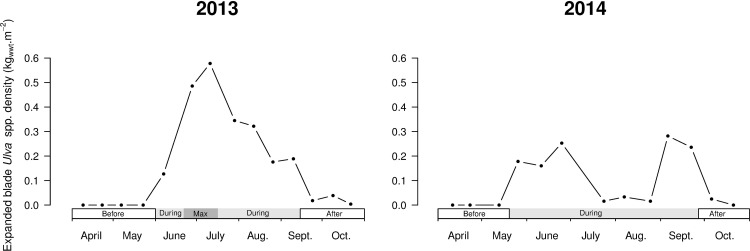
*Ulva* spp. densities (in wet weight kg.m^-2^) at the impacted site during the sampling period in 2013 and 2014. On each graph, the periods of the green tide are marked on the x-axis: *Before*, *During* and *After*. A *Max* period was added to the 2013 proliferation to underline the high peak of the green tide.

At the impacted site, green tides lasted (Fig 2) from early June to early September in 2013 (average: 0.3 kg_wwt_.m^-2^; maximum: 0.6 kg_wwt_.m^-2^ in July) and from the end of May to the end of September in 2014 (average: 0.1 kg_wwt_.m^-2^; two maxima: 0.26 kg_wwt_.m^-2^ in June and 0.3 kg_wwt_.m^-2^ in September). The green tides could be defined as medium in 2013 and low in 2014 with respect to the levels of *Ulva* spp. proliferation in this sandy beach area for the two last decades [22]. Other than these green macroalgae proliferations, no submerged aquatic vegetation was encountered in significant density at the impacted site nor at the control site [22].

The annual cycle of green macroalgae proliferation allowed for the delineation of three periods according to the presence of *Ulva* spp. at the impacted site: *Before*, *During* and *After* proliferation (Fig 2). The period of maximum green macroalgae density, from mid-June to mid-July 2013, with densities greater than 0.35 kg_wwt_.m^-2^, was considered as an additional “*Max”* stage (Fig 2). The densities of green macroalgae during proliferation were characterised as low: below 0.2 kg_wwt_.m^-2^, medium: between 0.2 and 0.35 kg_wwt_.m^-2^, and high: greater than 0.35 kg_wwt_.m^-2^.

### Habitat signature– δ^13^C

**Fig 3:**
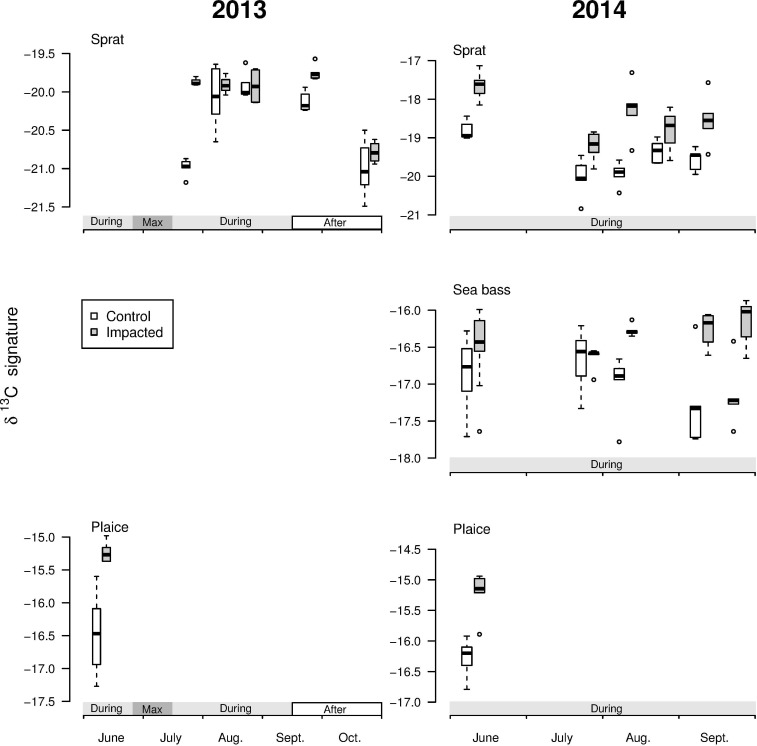
δ^13^C signatures (in ‰) for sprat (*S*. *sprattus*), sea bass (*D*. *labrax*) and plaice (*P*. *platessa*) at the control and impacted sites for the selected dates in 2013 and 2014. On each graph, the periods of the green tide are marked on the x-axis (see Fig 2).

In 2013, the δ^13^C signatures of plaice were higher at the impacted site, but the δ^13^C signatures of sprat were not significantly different between sites (Fig 3 and Table 2). In 2014, the δ^13^C signatures of sprat, sea bass and plaice were significantly higher at the impacted site (Fig 3 and Table 2).

**Table 2:** Statistical results (*p* values: ‘***’ <0.001; ‘**’ <0.01; ‘*’ <0.05) of the δ^13^C signature, TEAC, C:N ratio and R morphometric condition analyses for sprat (*S*. *sprattus*), sea bass (*D*. *labrax*) and plaice (*P*. *platessa*) in 2013 and 2014. ANOVA type: one-way ANOVA when indices were assessed on a single date and two-way ANOVA when several dates were analysed (factors “site”, “date” and their interaction “site:date”). When an index response was significantly influenced by the factor “site”, a comparison between the control (C) and impacted (I) sites was added.

Type of analysis	Species	Year	ANOVA type	Site	Date	Interaction
δ^13^C signature	Sprat	2013	two-way	<0.001 *** C < I	<0.001 ***	<0.001 ***
		2014	two-way	<0.001 *** C < I	<0.001 ***	0.146
	Sea bass	2014	two-way	<0.001 *** C < I	0.885	0.121
	Plaice	2013	one-way	0.003 ** C < I		
		2014	one-way	<0.001 *** C < I		
TEAC	Sprat	2013	one-way	0.328		
		2014	one-way	0.508		
	Sea bass	2014	one-way	0.301		
	Plaice	2014	one-way	0.001** C < I		
C:N ratio	Sprat	2013	two-way	0.504	<0.001 ***	0.024 *
		2014	two-way	0.016 * C > I	0.167 *	0.337
	Sea bass	2014	two-way	0.361	0.919	0.496
	Plaice	2013	one-way	0.050		
		2014	one-way	0.448		
R morphometric	Sprat	2013	two-way	0.346	<0.001 ***	0.005 **
condition		2014	two-way	0.225	<0.001 ***	<0.001 ***
	Sea bass	2014	two-way	<0.001 *** C < I	<0.001 ***	<0.001 ***
	Plaice	2013	one-way	0.332		
		2014	one-way	<0.001 *** C < I		

### Fish physiological responses to green tides

#### Fish instantaneous response–antioxidant defence capacity

Fish TEAC was examined at a medium density of *Ulva* spp. on 29 July 2013 and 6 June 2014 (Fig 2 and S1 Fig). For sprat and sea bass, no difference in antioxidant defence capacity was recorded between sites (Table 2). Conversely, on 6 June 2014, the antioxidant defence capacity of juvenile plaice at the impacted site was significantly higher than at the control site (Table 2 and S1 Fig).

#### Short-term response–muscle total lipid

Muscle total lipid measured using the C:N ratios did not differ between sites for all the species, except for sprat in 2014 (Table 2 and S2 Fig). In 2013, the C:N ratios of sprat increased at both sites from the end of July to the end of August, while in 2014, this increase only occurred at the control site. The C:N ratios of sprat remained stable and thus lower at the impacted site than at the control site in 2014.

#### Mid-term response–morphometric condition

For the three species, significant inter-individual morphometric condition variability was found at both the control and impacted site during the two studied annual cycles (S3 Fig). Fish condition did not differ between sites in 2013. In 2014, at the beginning of the green tide, i.e., at a low density of green macroalgae, plaice were in better condition at the impacted site (Table 2 and S3 Fig). For sprat and sea bass, a similar pattern was observed in 2014 during early green tide proliferation in spring, but it did not persist in summer (S3 Fig). For each studied year, no significant pattern in fish condition was revealed for these two species (Table 2).

#### Response throughout the juvenile period–daily growth rate

In 2013, no fish were present at the impacted site during the maximum of the green tide from mid-June to mid-July (period *Max* in Fig 2;[22]). The back-calculated DGRs during this period were thus excluded from the analysis.

**Fig 4:**
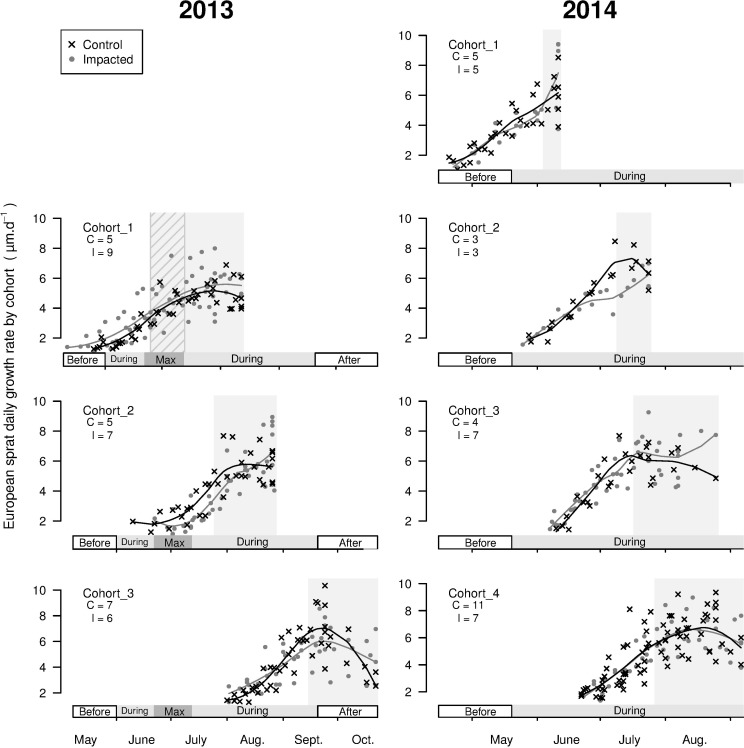
Otolith daily growth rate (μm.d^-1^) of juvenile sprat (*S*. *sprattus*) at the control and impacted sites for each cohort in 2013 and 2014, represented by a 10-day interval. Lines: associated local polynomial regression fitting (span = 0.7) of the cohort DGRs by site. On each graph, the periods of the green tide are marked on the x-axis (see Fig 2). Grey backgrounds: the juvenile period of the cohort (i.e., from settlement until capture). Hatched background: period during which no fish were captured at the impacted site (DGRs of this period were excluded from the growth rate analysis). The cohort name and number of fish analysed at the control (C) and impacted (I) sites are reported on the upper left part of each graphic.

For sprat, three cohorts were identified in 2013 and four in 2014. These cohorts settled throughout the green macroalgae proliferation period, from June until September (Fig 4). The DGRs of sprat did not differ between sites in 2013 nor in 2014, except for cohort 2 in 2014. For this cohort, which settled in sandy beaches at medium densities of green macroalgae in early July (i.e., just after the first maximum of *Ulva* spp. density), the DGRs were significantly lower at the impacted site than at the control site (Fig 4 and Table 3).

**Table 3:** ANOVA results (*p* values: ‘***’ <0.001; ‘**’ <0.01; ‘*’ <0.05) of the juvenile daily growth rate analysis for sprat (*S*. *sprattus*), sea bass (*D*. *labrax*) and plaice (*P*. *platessa*) in 2013 and 2014. Green tide period: period of the green tide cycle at the impacted site (see Fig 2). Cohorts: group of fish according to their date of first increment (see Figs 4–6). ANOVA type: one-way ANOVA when indices were assessed on a single date and two-way ANOVA when several dates were analysed (factors “site”, “date” and their interaction “site:date”). When the daily growth rate was significantly influenced by the factor “site”, a comparison between control (C) and impacted (I) sites was added.

Species	Year	Cohort	Green tide eriod	ANOVA	Site	Date	Interaction
			period	type			
Sprat	2013	Cohort_1	*During*	two-way	0.176	0.997	0.966
		Cohort_2	*During*	two-way	0.539	0.558	0.887
		Cohort_3	*During*	two-way	0.089	0.075	0.723
			*After*	two-way	0.799	0.001 **	0.062
	2014	Cohort_1	*During*	one-way	0.186		
		Cohort_2	*During*	two-way	0.010 * C > I	0. 512	0.019 *
		Cohort_3	*During*	two-way	0.160	0.119	0.052
		Cohort_4	*During*	two-way	0.520	0.017 *	0.500
Sea bass	2014	Cohort_1	*During*	two-way	<0.001 *** C > I	<0.001 ***	0.971
Plaice	2013	Cohort_1	*Before*	two-way	0.845	0.814	0.497
			*During*	one-way	0.009 ** C > I		
	2014	Cohort_1	*Before*	two-way	0.165	0.295	0.630
			*During*	two-way	0.05 * C > I	0.472	0.45
		Cohort_2	*Before*	two-way	0.006 ** C < I	0.1	0.257
			*During*	two-way	0.018 * C < I	0.222	0.607

**Fig 5:**
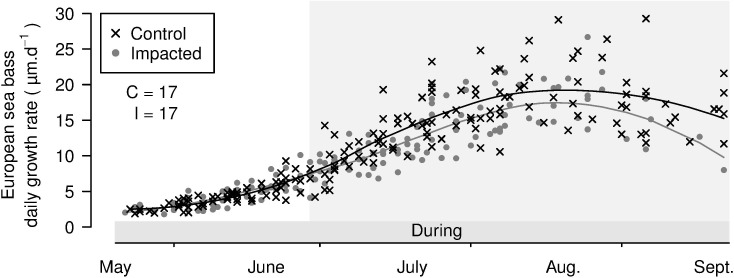
Otolith daily growth rate (μm.d^-1^) of juvenile sea bass (*D*. *labrax*) at the control and impacted sites in 2014, represented by a 10-day interval. Lines: associated local polynomial regression fitting (span = 0.7) of the cohort DGRs by site. On each graph, the periods of the green tide are marked on the x-axis (see Fig 2). Grey backgrounds: the juvenile period of the cohort (i.e., from settlement until capture). The number of fish analysed at the control (C) and impacted (I) sites is reported on the upper left part of the graphic.

Only one cohort of sea bass was identified in 2014. The cohort settled at the end of June at medium densities of green macroalgae (i.e., during the first maximum of *Ulva* spp. density in 2014). The DGRs of sea bass were significantly lower at the impacted site than at the control site (Fig 5 and Table 3).

**Fig 6:**
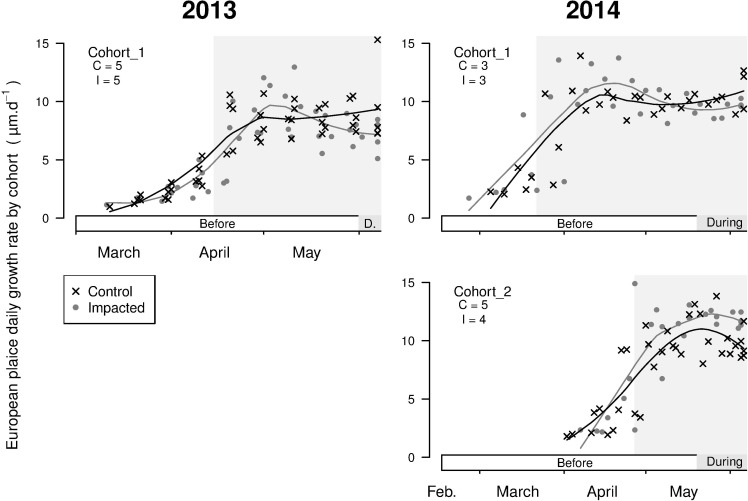
Otolith daily growth rate (μm.d^-1^) for juvenile plaice (*P*. *platessa*) at the control and impacted sites for each cohort in 2013 and 2014, represented by a 10-day interval. Lines: associated local polynomial regression fitting (span = 0.7) of the cohort DGRs by site. On each graph, the periods of the green tide are marked on the x-axis (see Fig 2). Grey backgrounds: the juvenile period of the cohort (i.e., from settlement until capture). The cohort name and number of fish analysed at the control (C) and impacted (I) sites are reported on the upper left part of each graphic.

For plaice, one cohort in 2013 and two in 2014 were identified. These three cohorts settled at least one month before the beginning of the green tides (Fig 6), which enabled the analysis of two periods of growth for plaice: *before* and *during* green tides (Table 3). Before green tides, the DGRs of juvenile plaice did not differ between sites for the cohort established in 2013 nor for cohort 1 in 2014. However, cohort 2 in 2014, which settled in late April, had significantly higher DGRs at the impacted site before green tides. At the onset of macroalgal proliferation, the plaice DGRs were significantly lower at the impacted site than at the control site for the cohort in 2013 and for cohort 1 in 2014. However, the plaice DGRs of cohort 2 in 2014 were still significantly higher at the impacted site from the onset of green tides onwards (Fig 6 and Table 3).

## Discussion

Under stressful conditions, juvenile fish implement biochemical, physiological and behavioural coping responses. These aim to first accommodate the disturbance and maintain fish homeostasis and then to limit their exposure to perturbation when conditions become detrimental [46]. Here, the responses of juvenile fish to green tides have been analysed on different integrative scales on three fish species, at both control and impacted sandy beaches. Considering the responses of the studied behavioural and physiological indices, a predominant negative influence of green tides on juvenile fish was demonstrated, with species-specific sensitivity to the perturbed conditions. A decreasing gradient of sensitivity to green tides was highlighted from benthic species, the first and most deeply affected, to demersal and pelagic fish species, which were less affected. Fish responses escalated with an increase in green macroalgae density, from the implementation of an instantaneous physiological response when environmental conditions are perceived as a perturbation, to adjustments in growth and muscle total lipids, and finally the disappearance of the fish species from the impacted site.

### Habitat selection

When changes in habitat suitability are induced by green tides, fish can die, emigrate [20], both resulting in a decrease in fish density [22], or remain in the disturbed habitat. Habitat selection was examined using the fish species catch records during the standardised sampling survey (S1 Table; [22]) and their habitat signatures from fish δ^13^C signature. The habitat selection of each species was strongly influenced by the density of green macroalgae.

At low densities of green macroalgae (i.e., at the beginning of green tides), juveniles of the three studied species were captured at both sites. For each species, δ^13^C signatures supported their site fidelity (i.e., higher signatures at the impacted site; see the methods section). Patchy cover of macroalgae at low densities for restricted periods of time therefore had no noticeable negative impact on the habitat selection of juvenile fish [22,75].

When medium densities of green macroalgae were reached, plaice disappeared from the impacted site, whereas they were still captured at the control site. Only one plaice was captured each year in September when the density of green macroalgae was decreasing. The disappearance of a fish species in the catches records could be considered as a result of avoidance behaviour [76] and/or local mortality at the impacted site. Even if plaice could avoid vegetated or clogged substrates when alternative substrates are locally available [77–79], both its weak capacity to migrate [42] and the drastic reduction of its abundance at the impacted site suggest plaice mortality occurred within areas impacted by green tides [20]. In contrast, at medium densities of green macroalgae, sprat and sea bass were captured at each site during both years. When medium densities of green macroalgae were maintained over the seasonal proliferation, as in 2014, their habitat signatures (i.e. δ^13^C signatures) remained different between sites throughout the surveyed period. This underlined the fidelity of these species to their site of capture and its vicinity.

When high densities of green macroalgae were reached, sprat and sea bass were not captured at the impacted site. The great mobility of sprat [53] and sea bass [80] could have allowed them to escape from the disturbed area during massive proliferations. Furthermore, in 2013, at medium densities of green macroalgae following the high densities, the δ^13^C signatures of sprat were similar across the control and impacted sites. Given the turnover rates for δ^13^C [32,51–53], this similarity indicates that sprat juveniles were not resident at the impacted site during the maximum proliferation but immigrated recently. At the same time, sea bass were also captured at the impacted site but at a density too low for analysis of habitat signature. This suggests that highly mobile species had returned to the impacted site after a significant decrease in green macroalgae density. Hence, both the green macroalgae density and the fish species capacity to move and to tolerate environmental pressures were driving factors of fish species habitat selection and fidelity. Here, sprat and sea bass implemented emigration, while mortality was the most probable scenario for plaice within the bay [20,77].

### Physiological responses of juvenile fish

#### Fish instantaneous responses

TEAC analysis under a medium density of *Ulva* species at the beginning of proliferation demonstrated an increase in fish antioxidant defence capacity for plaice. This increase in physiological defence reflects their perception of non-suitable conditions [46]. The accumulation of both fresh and senescent free-floating green macroalgae on the sea bottom [81] modifies the conditions of the benthic habitat [15,82,83] as soon as *Ulva* species proliferate at medium density. This, combined with the behaviour and relatively weak mobility of juvenile plaice [42,84], may explain the early impact of green tides on the benthic plaice and the lack of response of the demersal sea bass and the pelagic sprat, that are more mobile species [53,80].

#### Fish short-term and mid-term responses

An increase in muscle total lipid was observed for sprat from the end of July to the end of August at both sites in 2013 and only at the control site in 2014. This pattern could be explained by the increase of structural and storage lipids in fish muscle during favourable environmental conditions in summer [85,86]. In contrast, this increase in muscle total lipids was not observed for sprat at the impacted site under medium densities of green macroalgae in 2014. This difference in response of sprat between years could be attributed to its habitat fidelity to the impacted site under medium densities of green macroalgae in 2014 and not in 2013. During periods of stress and starvation, muscle total lipid could be reduced for each of the studied species [87–89]. However, the main lipid storage is in the liver for plaice and sea bass, and it is less important in the white muscle for these species than for sprat [86,90]. These species differences in main lipid storage localisation are a possible cause of the absence of a response of plaice and sea bass muscle total lipid content to the perturbed habitat conditions.

Somatic fish health status was analysed using the R morphometric condition index. Compared to the control site, better body condition was observed for all fish species at the impacted site under a low density of green macroalgae in 2014. This positive influence of low patchy green macroalgae proliferation might be explained by the local increase in new shelter and food resources [24–26]. Later in the proliferation of green macroalgae, no clear trend of beneficial or detrimental effects of green tides on fish condition was observed. The high inter-individual variability in the condition of each species may have masked the difference in individual fish conditions between sites. Moreover, considering the turnover rate of the morphometric condition index [62], fish may disappear from the impacted site, as a result of emigration or death [20], before the integration of a response to morphometric conditions, resulting in undetectable responses compared to daily growth and lipid indices.

The otoliths DGRs were examined to assess the individual growth history of juvenile fish. According to species-specific spawning season, the fish settled in intertidal areas at different periods of the green tide seasonal cycle. Sprat and sea bass settled at the studied sites during green tides. When settlement occurred at a medium density of green macroalgae, there was a negative influence of the green tides on the otolith growth rates of the settlers. Plaice settled at the sandy beaches before macroalgal blooms. At the beginning of the green tides, lower growth rates were recorded at the impacted site for the largest individuals but not for the smallest ones. These size-related differences in plaice response suggest a weaker influence of macroalgae on smaller individuals [27]. Alternatively, this differential response could arise from an estimation bias linked to differential mortality related to size in this species. A disproportionate mortality rate towards the smallest plaice caused by perturbed habitat conditions could lead to an overestimation of the growth rate of the younger individuals, selecting only faster growing individuals as survivors [6].

### Conclusions

Green tides affect fish habitat suitability through complex changes in biotic and abiotic conditions [17], including (i) changes in habitat structure of both the sea bottom and the water column [11]; (ii) changes in both the concentration and the daily amplitude of dissolved oxygen and the release of toxic substances (e.g., allelopathic substances and dimethyl sulphide) by fresh and senescent macroalgae, as well as by their bacterial flora [24,82,83,91]; and (iii) modifications in trophic conditions (e.g., density of available prey; [16,33,92,93]) as a result of (i) and (ii)). These changes in habitat conditions and their interactions [15,27,82] affect the individual performance of juvenile fish and their habitat selection. Fish responses to these modifications are characterised by an instantaneous response when environmental conditions are perceived as a perturbation, followed by adjustments to individual fish performance [94], especially fish lipids and growth, the two primary mechanisms of energy allocation of juvenile fish [85]. For the studied species, the decrease in muscle total lipid and fish growth rate in response to disturbed habitat conditions at medium density of green tides could be attributed to the reduction of both the feed intake and allocation of energy to growth and storage [20,82,95–97]. Then, under long-lasting and/or increasingly perturbed conditions, juvenile fish may implement avoidance behaviour [76], as observed for sprat, and/or a massive local increase in mortality, as suggested for plaice. According to species-specific tolerance, the capacity to move and the distribution in the water column, these responses are implemented at different densities of green macroalgae. These differences in response reveal a gradient of sensitivity to green tides from benthic species to demersal and pelagic fish species.

In light of fish species responses during green tides, habitats impacted by this perturbation are less suitable nursery grounds and become entirely unsuitable during massive proliferation [22]. This raises questions regarding the quantification of the effects of green tides on fish recruitment on the scale of their proliferation area. At a regional scale, the effect of this anthropogenic perturbation on fish recruitment could affect population dynamics and fisheries.

## Supporting Information

S1 Fig Trolox equivalent antioxidant capacity (TEAC; in mM Trolox equivalent/mg of soluble protein) for sprat (*S*. *sprattus*), sea bass (*D*. *labrax*) and plaice (*P*. *platessa*) at the control and impacted sites on 2013-07-29 and 2014-06-06. (TIF)

S2 Fig C:N ratio for sprat (*S*. *sprattus*), sea bass (*D*. *labrax*) and plaice (*P*. *platessa*) at the control and impacted sites for the selected dates in 2013 and 2014. On each graph, the periods of the green tide are marked on the x-axis (see Fig 2).
(TIFF)

S3 Fig R morphometric conditions for sprat (*S*. *sprattus*), sea bass (*D*. *labrax*) and plaice (*P*. *platessa*) at the control and impacted sites for the selected dates in 2013 and 2014. On each graph, the periods of the green tide are marked on the x-axis (see Fig 2).
(TIF)

S1 Table Total catches (number of individuals) for sprat (*S*. *sprattus*), sea bass (*D*. *labrax*) and plaice (*P*. *platessa*) at the control and impacted sites in 2013 and 2014 for each sampled date during the standardised sampling survey. Dates highlighted in grey correspond to the period during green tides, and the two dates highlighted in dark grey identify the maximum of the green tide in 2013. The underlined date shows the total catches during the 24 h survey. The two dates with a star indicate the number of supplementary fish captured for the analysis of fish antioxidant defence capacity. Bold total catches refer to the dates selected for the analysis at the individual scale for each species.
(DOCX)

S2 Table Linear regressions (*p* values: ‘***’<0.001; ‘**’<0.01; ‘*’<0.05) of the C:N basal signatures according to fish size for sprat (*S*. *sprattus*), sea bass (*D*. *labrax*) and plaice (*P*. *platessa*). (DOCX)

S3 Table Log length-mass equations by species and by year calculated by linear regression (*p* values: ‘***’<0.001) and used for the R morphological condition index. (DOCX)
