# Supplementary figures and images for: Influence of Green Tides in Coastal Nursery Grounds on the Habitat Selection and Individual Performance of Juvenile Fish
Source: PLoS One. 2017 Jan 26;12(1):e0170110. doi: 10.1371/journal.pone.0170110 (PMC5268461; doi:10.1371/journal.pone.0170110)

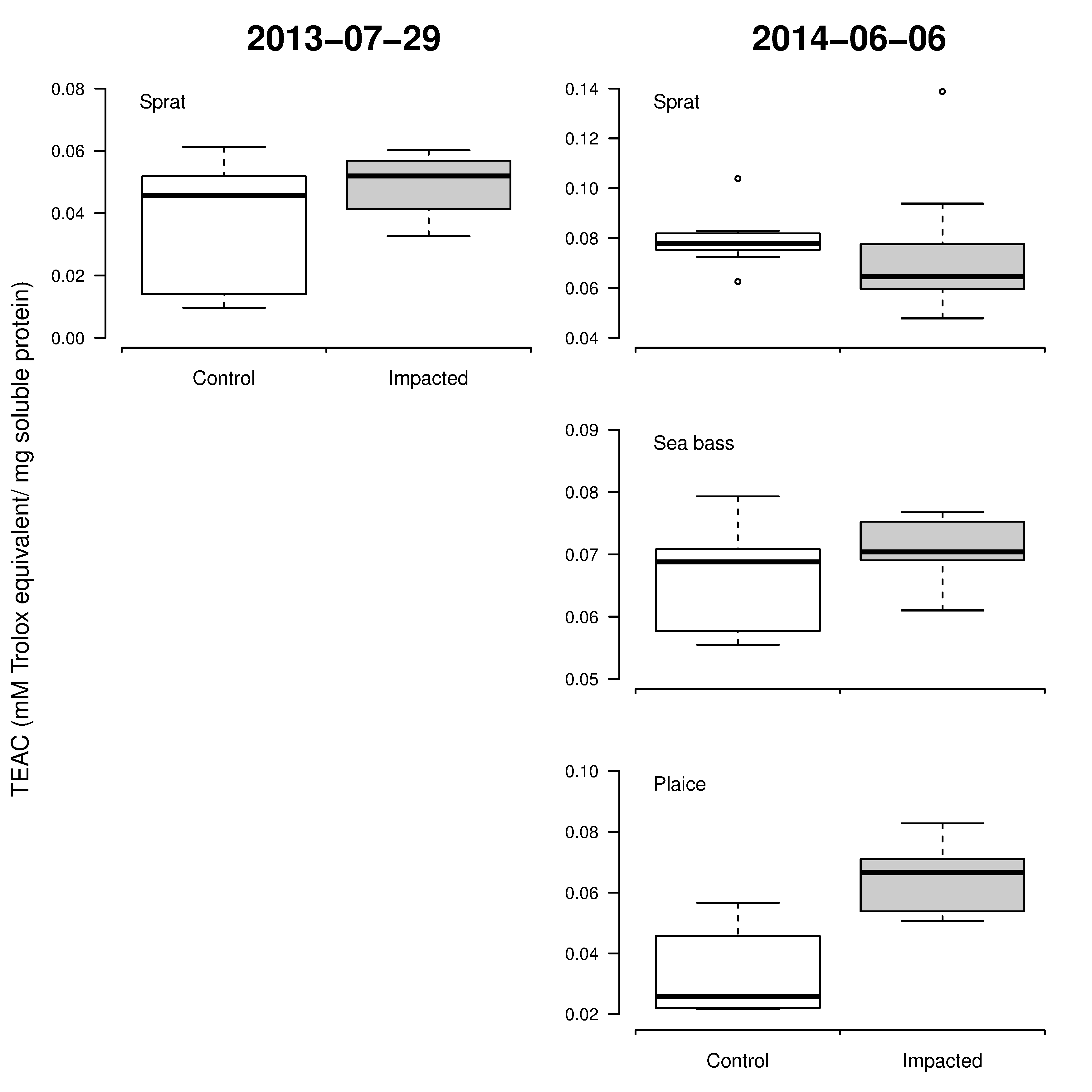

Supplement: S1 Fig — (TIF) [file pone.0170110.s001.tif]

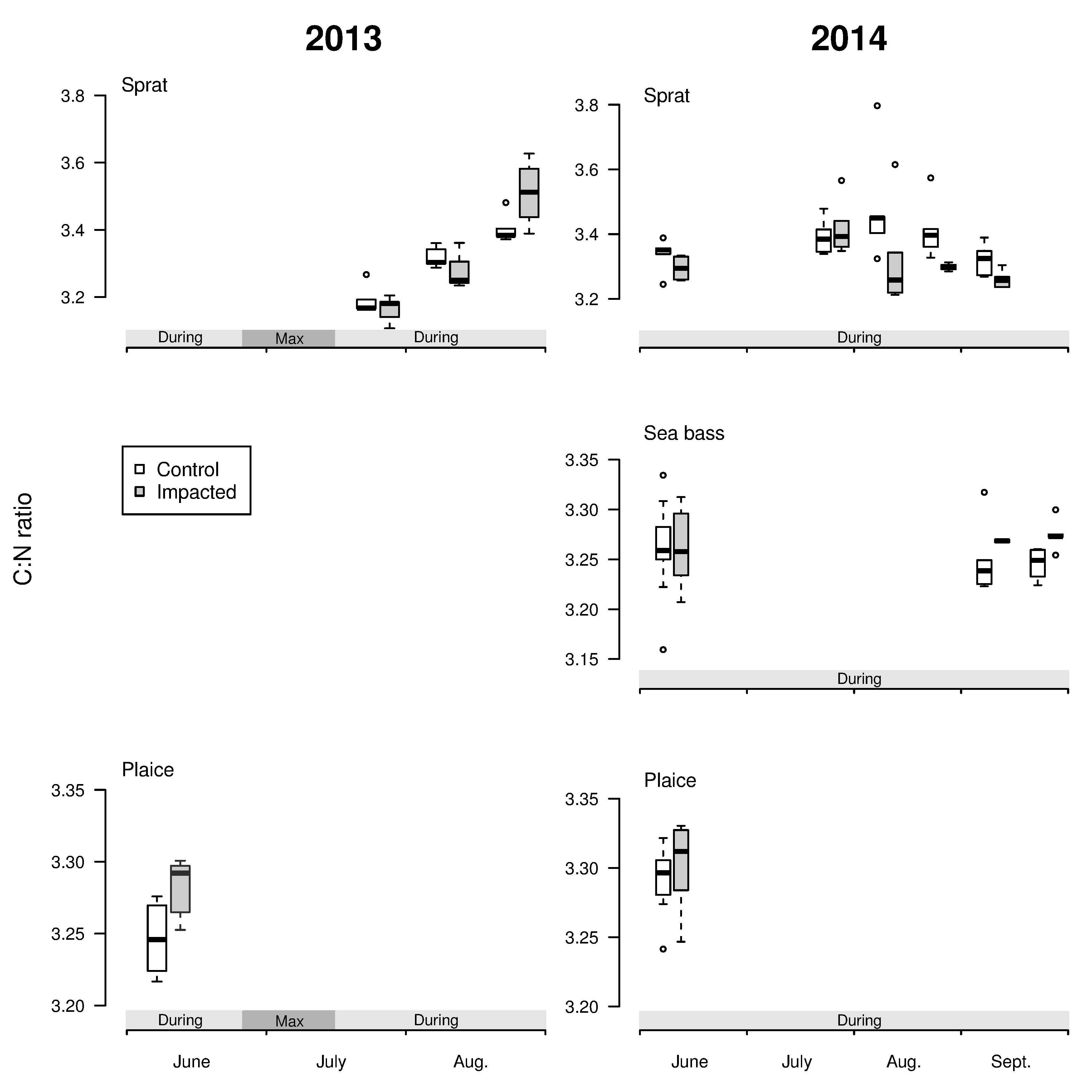

Supplement: S2 Fig — On each graph, the periods of the green tide are marked on the x-axis (see Fig 2). (TIFF) [file pone.0170110.s002.tiff]

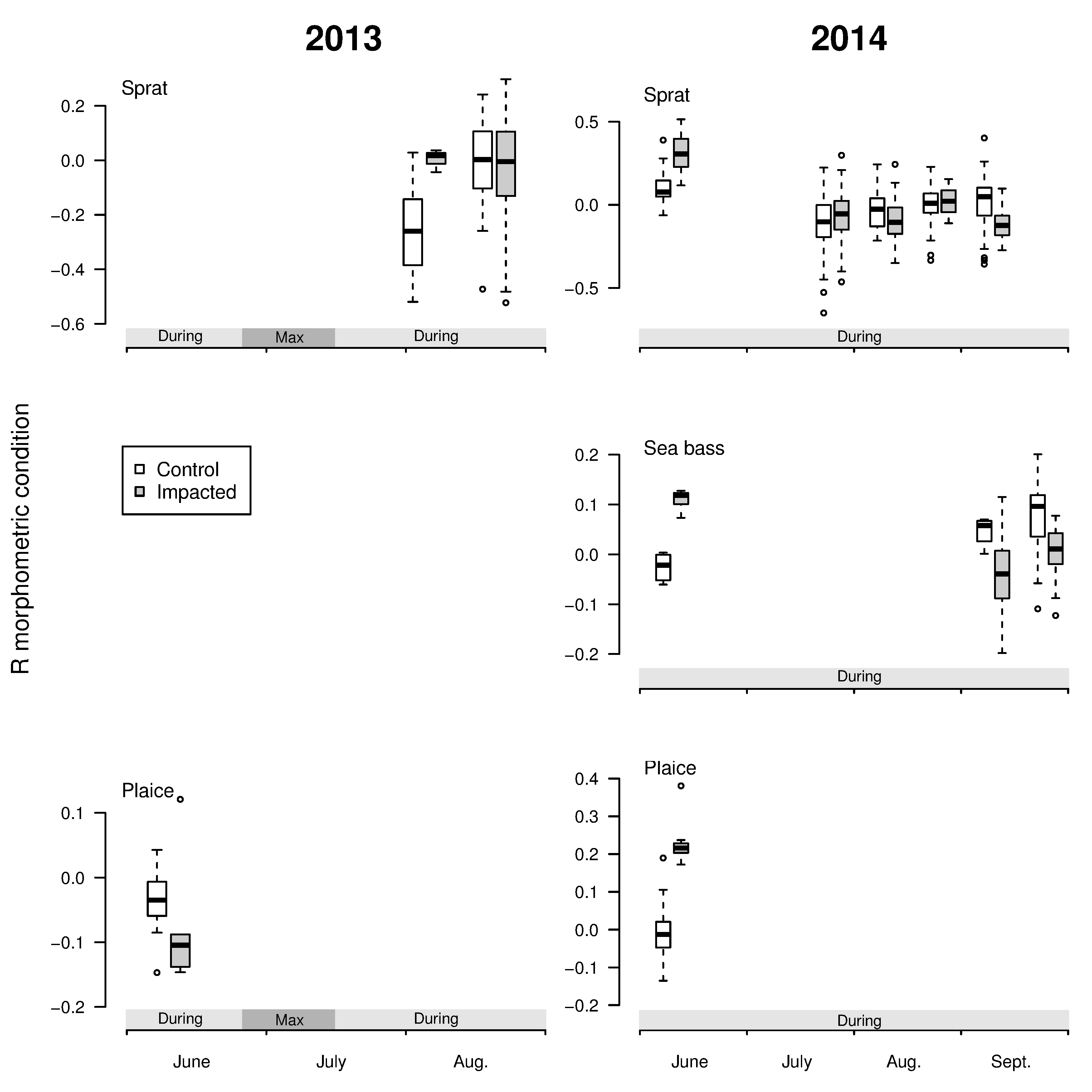

Supplement: S3 Fig — On each graph, the periods of the green tide are marked on the x-axis (see Fig 2). (TIF) [file pone.0170110.s003.tif]
