# Supplementary material for: Influence of Green Tides in Coastal Nursery Grounds on the Habitat Selection and Individual Performance of Juvenile Fish
Source: PLoS One. 2017 Jan 26;12(1):e0170110. doi: 10.1371/journal.pone.0170110 (PMC5268461; doi:10.1371/journal.pone.0170110)
Supplement: S1 Table — Dates highlighted in grey correspond to the period during green tides, and the two dates highlighted in dark grey identify the maximum of the green tide in 2013. The underlined date shows the total catches during the 24 h survey. The two dates with a star indicate the number of supplementary fish captured for the analysis of fish antioxidant defence capacity. Bold total catches refer to the dates selected for the analysis at the individual scale for each species. (DOCX) [file pone.0170110.s004.docx]

**S1 Table**. **Total catches (number of individuals) for sprat (*S. sprattus*), sea bass (*D. labrax*) and plaice (*P. platessa*) at the control and impacted sites in 2013 and 2014 for each sampled date during the standardised sampling survey.** Dates highlighted in grey correspond to the period during green tides, and the two dates highlighted in dark grey identify the maximum of the green tide in 2013. The underlined date shows the total catches during the 24 h survey. The two dates with a star indicate the number of supplementary fish captured for the analysis of fish antioxidant defence capacity. Bold total catches refer to the dates selected for the analysis at the individual scale for each species.

|  |  | **Sprat** | | **Sea bass** | | **Plaice** | |
| --- | --- | --- | --- | --- | --- | --- | --- |
| **Year** | **Date** | **Control** | **Impacted** | **Control** | **Impacted** | **Control** | **Impacted** |
| 2013 | 2013-04-09 |  |  |  |  |  |  |
|  | 2013-04-23 |  |  |  |  |  |  |
|  | 2013-05-07 |  |  |  |  |  |  |
|  | 2013-05-23 |  |  |  |  | 1 | 2 |
|  | 2013-06-07 |  | 46 |  |  | **9** | **6** |
|  | 2013-06-28 |  |  |  |  | 231 |  |
|  | 2013-07-11 |  |  |  |  | 128 |  |
|  | 2013-07-29 |  | 15 |  |  |  |  |
|  | *2013-07-29 ** | ***5*** | ***5*** |  |  |  |  |
|  | 2013-08-12 | **44** | **3** | 3 | 1 | 85 |  |
|  | 2013-08-26 | **80** | **87** | 4 | 1 | 35 |  |
|  | 2013-09-10 |  | 4 | 3 |  | 12 | 1 |
|  | 2013-09-24 | **1412** | **820** | 3 | 8 | 1 |  |
|  | 2013-10-09 | 2 |  |  | 2 | 7 |  |
|  | 2013-10-22 | **343** | **57** |  |  | 10 |  |
|  | **Total 2013** | 1886 | 1037 | 13 | 12 | 519 | 9 |
| 2014 | 2014-04-11 |  |  |  | 2 | 2 | 2 |
|  | 2014-04-23 | 199 |  | 2 | 8 | 1 |  |
|  | 2014-05-13 |  |  |  |  |  |  |
|  | 2014-05-26 | 35 | 1 |  |  |  |  |
|  | 2014-06-06 | 5 | 1 | **9** | **7** | **36** | **13** |
|  | *2014-06-06 ** | ***10*** | ***11*** | ***8*** | ***6*** | ***15*** | ***9*** |
|  | 2014-06-11 | **15** | **31** |  |  | 3 |  |
|  | 2014-06-25 |  | 1 |  |  | 6 |  |
|  | 2014-07-24 | **759** | **259** | **88** | **43** | 12 |  |
|  | 2014-08-07 | **15** | **152** | **79** | **110** | 3 |  |
|  | 2014-08-25 | **644** | **3** | **4** | **57** |  |  |
|  | 2014-09-06 | **296** | **205** | **13** | **16** | 3 |  |
|  | 2014-09-22 | 719 |  | **14** | **14** |  | 1 |
|  | 2014-10-06 | 284 |  | **3** | **40** |  |  |
|  | 2014-10-21 |  |  | 1 | 29 |  |  |
|  | **Total 2014** | 2981 | 664 | 221 | 332 | 81 | 25 |
|  | **Total** | 4867 | 1701 | 234 | 344 | 600 | 34 |
