# Supplementary material for: Influence of Green Tides in Coastal Nursery Grounds on the Habitat Selection and Individual Performance of Juvenile Fish
Source: PLoS One. 2017 Jan 26;12(1):e0170110. doi: 10.1371/journal.pone.0170110 (PMC5268461; doi:10.1371/journal.pone.0170110)
Supplement: S2 Table — (DOCX) [file pone.0170110.s005.docx]

**S2 Table. Linear regressions (*p* values: ‘***’<0.001; ‘**’<0.01; ‘*’<0.05) of the C:N basal signatures according to fish size for sprat (*S. sprattus*), sea bass (*D. labrax*) and plaice (*P. platessa*).**

| **Species** | **Df** | **MS** | **F value** | ***p*** |
| --- | --- | --- | --- | --- |
| Sprat | 1 | 0.009806 | 0.537 | 0.475 |
| Residuals | 15 | 0.018246 |  |  |
| Sea bass | 1 | 0.0007029 | 1.103 | 0.313 |
| Residuals | 13 | 0.0006370 |  |  |
| Plaice | 1 | 0.0010118 | 9.249 | 0.0558 |
| Residuals | 3 | 0.0001094 |  |  |
