# Supplementary material for: Influence of Green Tides in Coastal Nursery Grounds on the Habitat Selection and Individual Performance of Juvenile Fish
Source: PLoS One. 2017 Jan 26;12(1):e0170110. doi: 10.1371/journal.pone.0170110 (PMC5268461; doi:10.1371/journal.pone.0170110)
Supplement: S3 Table — (DOCX) [file pone.0170110.s006.docx]

**S3 Table. Log length-mass equations by species and by year calculated by linear regression (*p* values: ‘***’<0.001) and used for the R morphological condition index.**

| **Species** | **Years** | **Formula** | **r²** | ***p*** |
| --- | --- | --- | --- | --- |
| Sprat | 2013 | Log(W)= (3.954010*log(L))-8.825545 | 0.8234 | <0.001*** |
|  | 2014 | Log(W)= (3.558647*log(L))-7.279420 | 0.9271 | <0.001*** |
| Sea bass | 2014 | Log(W)= (2.998706*log(L))-4.591881 | 0.994 | <0.001*** |
| Plaice | 2013 | Log(W)= (2.993046*log(L))-4.496225 | 0.99 | <0.001*** |
|  | 2014 | Log(W)= (2.916018*log(L))-4.117287 | 0.9909 | <0.001*** |
